# Optical Coherence Elastography-Based Corneal Strain Imaging During Low-Amplitude Intraocular Pressure Modulation

**DOI:** 10.3389/fbioe.2019.00453

**Published:** 2020-01-31

**Authors:** Sabine Kling, Hossein Khodadadi, Orcun Goksel

**Affiliations:** OPTIC Team, Computer-assisted Applications in Medicine Group, Computer Vision Laboratory, Department of Information Technology and Electrical Engineering, ETH Zurich, Zurich, Switzerland

**Keywords:** elastography, corneal biomechanics, optical coherence tomography, intraocular pressure, natural stress condition

## Abstract

**Purpose:** Optical coherence elastography (OCE) is a promising technique for high-resolution strain imaging in ocular tissues. A major strain-inducing factor in the eye is intraocular pressure (IOP), with diurnal physiological fluctuations reaching up to 5 mmHg. We study herein low-amplitude IOP modulation to assess local corneal strain patterns.

**Methods:** *Ex vivo* porcine eye globes were adjusted to an initial IOP of 15 mmHg and subsequently 25 mmHg. Corneal strain was induced by two subsequent pressure cycles, in which IOP was first increased and then decreased, each by a total of 5 mmHg. Two-dimensional optical coherence tomography (2D-OCT) B-scans were recorded after each loading step. Axial strain maps were obtained from magnitude and phase changes and supra-pixel displacements from cross-correlation. The strain detection sensitivity was evaluated in an isotropic material.

**Results:** Deformations arising from a single 1-mmHg step could be resolved. The largest strain amplitudes (5.11·10^−3^) were observed in the posterior stroma at a low initial IOP. Strain amplitude was 1.34 times higher at 15 mmHg than at 25 mmHg (*p* = 0.003). Upon IOP increase, the anterior cornea was compressed, whereas the posterior cornea showed axial expansion. Both morphological images and strain maps were sensitive to postmortem time. Strains that are larger than 2.44·10^−5^ could be reliably measured.

**Conclusions:** Low-amplitude IOP modulation, similar to diurnal physiological changes, induced measurable deformations in corneal tissue. Axial strain maps permit a localized comparison of the corneal biomechanical response. Small-strain OCE can likely be extended to other domains.

## Introduction

Intraocular pressure (IOP) is the principal source of mechanical stress in ocular tissues. IOP undergoes diurnal physiologic fluctuations of ~5 mmHg in healthy individuals, with highest values in the first half of the day (David et al., 1992; Wilensky et al., 1993). In glaucomatous eyes, in addition to a higher absolute IOP, its diurnal fluctuation is also higher (5.8 mmHg in open-angle glaucoma, 6.8 mmHg in ocular hypertension) (David et al., 1992). This means that ocular tissues are subjected daily to a low-amplitude variation of quasi-static mechanical loading.

Dynamic mechanical loading in combination with stress–strain analysis is also required for material characterization and hence of interest for the diagnosis and follow-up of degenerating ocular pathologies, such as keratoconus (Andreassen et al., 1980) and iatrogenic ectasia. In contrast to diurnal IOP fluctuations, air-puffs used to deform corneal tissue for subsequent geometrical analysis subject the eye to a much higher mechanical stress (Kling et al., 2014) (~110 mmHg, 30 ms duration). While such high mechanical loads lead to a large macroscopic deformation, it is questionable how clinically relevant such measurements are—this because collagen fibers in the anterior surface relax during inward motion and hence do not contribute to load bearing (Ariza-Gracia et al., 2015). In addition, a large proportion of displacement is due to bulk motion inducing strong dependency on geometry. Most importantly, standard air-puff-based techniques (Shah et al., 2007; Vinciguerra et al., 2016) only provide parameters describing deformation characteristics of the entire eye and do not allow lateral or axial spatial resolution. Recently, micro-air-puff stimulation in combination with shear wave propagation imaging was proposed using optical coherence elastography (OCE) (Wang and Larin, 2014). As this approach applies relatively small and short (1 Pa, ~1 ms duration) mechanical loads, it measures dynamic tissue properties. While dynamic measurements allow fast imaging speeds and hence reduce motion artifacts, longer time scales, which are relevant for corneal reshaping and disease progression, are not accessible. The only quasi-static assessment of *in vivo* corneal biomechanics combines OCE with central applanation of the cornea by a gonioscopy lens (De Stefano et al., 2018) requiring contact. Here, spatially resolved cross-sectional maps of axial corneal displacements were obtained by tracking speckle deformation during the applanation (De Stefano et al., 2018).

The physiological stress distribution in the cornea arises from IOP and hence is tensile and compressive throughout the full stroma—with a gradient from largest stresses at the posterior surface toward lowest stresses at the anterior surface. In contrast to IOP-induced loading, corneal stress distribution during air-puff, and anterior applanation measurement designs is different: the anterior stroma is subjected to compression, whereas the posterior stroma is subjected to tensional stress (Ariza-Gracia et al., 2015). This unnatural and non-physiological stress distribution may render the interpretation of the retrieved elastic properties difficult and limit the relevance (Ariza-Gracia et al., 2015) with respect to clinical predictions, for example, on disease progression or surgical outcomes.

Eye inflation experiments have been applied in the past for *ex vivo* corneal mechanical characterization (Elsheikh and Anderson, 2005; Kling et al., 2010) and were shown to closely match results obtained from strip extensometry tests (Elsheikh and Anderson, 2005). A particular advantage over strip extensometry is that the collagen fibril network remains intact. However, when assessing corneal curvature changes (Kling et al., 2010) or apex displacement (Elsheikh and Anderson, 2005) alone, corneal properties cannot be clearly separated from deformation of other ocular components, including the sclera and crystalline lens.

Therefore, the purpose of this study was to analyze corneal strain distribution resulting from low-amplitude IOP modulation with high spatial resolution and under physiologic stress distribution, in particular with respect to the axial deformation and strain profile. In this context, axial direction refers to the optical axis of the imaging system. At the same time, the sensitivity of detecting axial strain shall be evaluated. Furthermore, this study shall permit a better understanding of the effect of different biases in ocular biomechanical assessments, such as IOP dependency and inhomogeneous strain distribution across the cornea. The hypothesis is that corneal strain maps arising from pressure changes similar to physiological IOP fluctuations can be retrieved with OCE imaging and be used for biomechanical interpretation.

## Materials and Methods

### Sample Preparation

**Figure 1:**
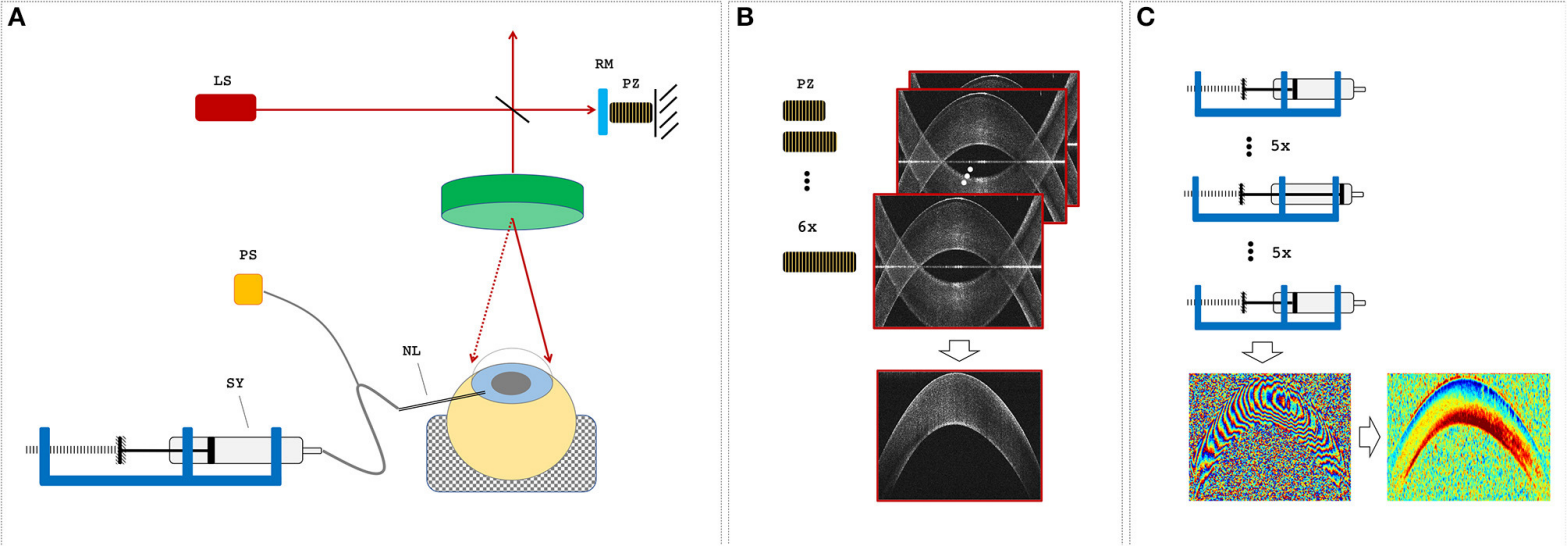
Measurement setup. **(A)** Optical coherence tomography (OCT) setup and sample alignment. LS, light source; RM, reference mirror; PZ, piezoelectric actuator; NL, needle; SY, syringe; PS, pressure sensor. **(B)** A single OCT measurement set consisted of six subsequent B-scans, during which the PZ was used to remove the mirror artifact by changing the reference arm length. **(C)** The pressure control unit was used to increase and then decrease the intraocular pressure (IOP) in steps of 1 mmHg before an OCT measurement set was recorded. Measurement sets at two distinct IOPs were used to determine phase difference and axial strain maps.

Six freshly enucleated porcine corneas of ~8 months of age and unknown gender were obtained from a local slaughterhouse where they underwent screening by a veterinarian to confirm the absence of any disease. Only eyes with transparent cornea and intact epithelium were used and measured within 9 h. Porcine eyes typically have a horizontal diameter of 14.3 mm, a vertical diameter of 12.0 mm, a corneal thickness of 1,001 μm (*ex vivo*)/666 μm (*in vivo*), a mean keratometry value of 40.0 diopters, and a diameter of 23.9 mm (Sanchez et al., 2011). Each eye was placed in the hemispherical recess (25 mm diameter) of a cylindrical silicone mold. A 20-gauge needle was inserted through the limbus into the anterior chamber and connected to a pressure control unit for IOP modulation (Figure 1A). The control unit consisted of a 5-ml syringe mounted on a rigid stage, a stepper motor (Can Stack Linear Actuator 35DBM-L, Portescap SA, La Chaux-de-Fonds, Switzerland), a pressure sensor (NPC-100, Amphenol Advanced Sensors, Pforzheim, Germany), and a closed-loop control program written in LabView (LabView 2016, National Instruments, Switzerland). Each eye was measured twice; the initial IOP was set to 15 mmHg in the first measurement cycle and to 25 mmHg in the second cycle. In each run, the IOP was successively increased and subsequently decreased by a total of 5 mmHg in steps of 1 mmHg every 12 s, corresponding to a 0.08-Hz frame rate for strain and displacement computation. At each pressure step, IOP was first adjusted by the pressure control unit, and subsequently, an OCT measurement set was recorded for deformation analysis (Figure 1C). Recording of a single pressure cycle consisting of 11 OCT scans with intermittent IOP adjustment took an average 154 s. Measurements were performed at room temperature (~21°C) and standard humidity (40–60%). Before starting each IOP cycle, corneal surface was humidified with a drop of PBS (Dulbecco's phosphate buffered saline; Sigma Aldrich, Switzerland). This was the only precaution taken to reduce corneal dehydration during the measurements. We used water as the liquid within the pressure system; this was considered unproblematic in terms of osmotic artifacts given that the injected volume was relatively small (~2 μl/step vs. ~140 μl anterior chamber volume) and the measurement duration was short.

### Imaging Protocol

A custom-built (Bern University of Applied Sciences, Switzerland) spectral domain optical coherence tomography (SD-OCT) system with a central wavelength of 878 nm and a bandwidth of 62.5 nm was used. Images were recorded with an A-line rate of 10 kHz, an integration time of 90 μs, and an intensity of 1.62 mW at the sample surface. The OCT system had an axial sampling precision of 4.48 μm, corresponding to its axial resolution in air, and a spot size of 12.5 μm, corresponding to its lateral resolution. All samples were imaged over a lateral range of 14 mm with 1,000 A-lines/B-scan. A single OCT measurement set consisted of six B-scans taken in series each with a size of 2,048 axial × 1,000 lateral pixels, whereas the length of the reference arm was modulated (Figure 1B) with a piezoelectric actuator in steps of 30 nm for full-range imaging (Zotter et al., 2010).

### Displacement Tracking and Strain Analysis

The raw SD-OCT signal was subjected to basic signal processing, including background removal, remapping, and dispersion compensation using procedures described earlier (Drexler and Fujimoto, 2008). The phase-shift induced by the 30-nm reference arm modulation was used to identify and suppress the mirror image inherent to spectral domain OCT images. For this purpose, a mask

(1a) $$\begin{array}{l} M\left(z,x\right)=\Delta \Phi_{\text{step}}>0 \end{array}$$

was generated and employed on the signal in spatial domain, where

(1b) $$\begin{array}{l} \Delta \Phi_{\text{step}}\left(z,x\right)=∠\overset{j_{\max}}{\underset{j\;=\;2}{\sum }}|A_{j}\left(z,x\right)|\cdot |A_{j-1}\left(z,x\right)|\\ \;\cdot \text{ exp}\left(-i\cdot ∠\left(A_{j-1}^{*}\left(z,x\right)\cdot A_{j}\left(z,x\right)\right)\right). \end{array}$$

Following the complex-valued vector summation approach described by Zaitsev et al. (2016) ΔΦ_step_ represents the magnitude-weighted phase difference between subsequent reference arm modulation steps at a given IOP. *A*(*z, x*):*z* ∈ ℕ_≤*m*_ × *x* ∈ ℕ_≤*n*_ → ℂ, with m = 2,048 and n = 1,000 representing the complex OCT signal in spatial domain, *z* and *x* indicating the axial and lateral positions, and ^*^ indicating the complex conjugate. *j* ∈ ℕ_≤_*j*__max__ indicates the number of steps for reference arm modulation with j_max_ = 6. Next, to compute the deformation induced by IOP modulation, the spatially resolved phase difference map ΔΦ_iop_ between the images of two subsequent pressure steps was determined using an approach similarly to that described above, with the difference that the summation was performed over a window of size 7 × 7 pixels with the purpose of noise removal and missing data fill-in. ΔΦ_iop_ corresponds to the angle of *C*_iop_(*z, x*) in the following equation:

(2a) $$\begin{array}{l} C_{\text{iop}}\left(z,x,p\right)=\overset{w_{z}}{\underset{j=-w_{z}}{\sum }}\overset{w_{x}}{\underset{k=-w_{x}}{\sum }}M\left(z+j,x+k\right)\cdot |A_{\text{iop}}\left(z+j,x+k\right)|\cdot |A_{\text{iop}+\Delta }\left(z+j+p,x+k\right)|\\ \;\cdot \text{exp}\left(-i\cdot ∠\left(A_{\text{iop}}^{*}\left(z+j,x+k\right)\cdot A_{\text{iop}+\Delta }\left(z+j+p,x+k\right)\right)\right)\text{ for}-3\leq p\leq 3 \end{array}$$

where *w*_z_ = 3 and *w*_x_ = 3 pixels. To quantify supra-pixel displacements (bulk motion), an axial grid search was performed over *p* and the magnitude of *C*_iop_ was used—similarly to standard cross-correlation speckle tracking—to extract macroscopic absolute axial supra-pixel displacements *z*_sp_ for aligning two subsequent B-scans during phase difference computation:

(2b) $$\begin{array}{l} \;\Delta z_{\text{sp}}\;=\;arg\max{\left\{\left|C_{\text{iop}}\left(z,x,p\right)\right|\right\}}_{p} \end{array}$$

Bulk motion compensation during processing is particularly necessitated for measurements with a low initial IOP. Note that bulk motion does not compromise strain analysis as long as displacement between two adjacent axial pixels is lower than λ/4, which corresponds to a strain of:

(2c) $$\begin{array}{l} \;\frac{\lambda_{\text{mean}}{\cdot n}_{\text{cornea}}}{4\cdot \text{asu}}\approx 6.7\%, \end{array}$$

where λ_mean_ is the central wavelength of the OCT (878 nm), *n*_cornea_ is the refractive index of corneal tissue (1.375), and asu is the axial sampling unit in air (4.48 μm). This strain threshold is more than 10 times larger than our experimentally measured strains. For enhanced interpretation of axial displacement, a parabola was fitted to the critical point Δ*z*_sp_ and its two neighbors (*p* = Δ*z*_sp_ ± 1) to determine subpixel shifts in addition to bulk motion. After correcting for bulk motion (i.e., evaluation at *p* = Δ*z*_sp_), the axial strain ε_z_ was determined from the axial gradient of ∠*C*_iop_ by summing the complex vector differences between the original image and a copy of it shifted by 1-pixel in z-direction, within a window of size 17 × 3 pixels, that is:

(3a) $$\begin{array}{l} \Delta \varepsilon_{\text{zz}}\left(z,x\right)=\;T\cdot \frac{\partial {∠C}_{\text{iop}}\left(z,x\right)}{\partial z}\\ \;=T\cdot ∠\sum_{j\;=\;-w_{z^{\prime }}}^{w_{z^{\prime }}}\sum_{i\;=\;-w_{x^{\prime }}}^{w_{x^{\prime }}}C_{\text{iop}}\left(z,x\right)\\ \;\cdot \;{C_{\text{iop}}}^{*}\left(z+1+j,x+k\right), \end{array}$$

with w_z_′ = 8 and w_x_′ = 1 pixels and

(3b) $$\begin{array}{l} T=\frac{\lambda_{\text{mean}}}{4\pi \cdot \text{asu}}, \end{array}$$

This complex processing approach can be understood as a weighted vector summation by the magnitude of the correlation. The angle of the summed vector across the processing window thus represents the mean phase shift and is little affected by pixels of low correlation (i.e., by noise). The same approach was applied to retrieve axial strain. In consequence, strain maps computed *via* Equation 3a were directly used for strain interpretation without any further post-processing. It should be noted that a phase unwrapping algorithm or a least-squares estimator is not required with this vector-based phase gradient approach (Zaitsev et al., 2016). The sizes of the processing windows for phase and strain maps were chosen to balance out noise levels (standard deviation) and image resolution (i.e., window size). For comparison, the size of the axial window (17 pixels) for our strain computation is similar to 16 pixels in a previous study (Zaitsev et al., 2016) using the same vector-based method. We centered the processing window on each pixel, that is, the window overlap was in all cases the window size minus one.

### Axial Strain Sensitivity

For a ground-truth controlled reference measurement of the error inherent to strain measurements in the current study, a homogeneous isotropic material [polydimethylsiloxan (PDMS)] with an elastic modulus of 200 kPa was stepwise laterally compressed while axial strain was measured by the OCT. Using the Cartesian coordinate system, Hook's law for an isotropic material (Bayoumi, 2018) can be expressed as σ = *E* · ε, where σ represents the stress tensor, ε the strain tensor, and *E* the stiffness tensor (or elastic modulus). Assuming plane stress is applied in the x-direction, the stress tensor can be reduced to:

(4a) $$\begin{array}{l} \sigma =\left[\begin{array}{ccc} \sigma_{\text{xx}} & 0 & 0\\ 0 & 0 & 0\\ 0 & 0 & 0 \end{array}\right]. \end{array}$$

The strain tensor then results in:

(4b) $$\begin{array}{l} \varepsilon =\left[\begin{array}{c} \varepsilon_{\text{xx},\text{GT}}\\ \varepsilon_{\text{yy},\text{GT}}\\ \varepsilon_{\text{zz},\text{GT}} \end{array}\right]=\left[\begin{array}{ccc} \frac{1}{E} & -\frac{\nu_{\text{PDMS}}}{E} & -\frac{\nu_{\text{PDMS}}}{E}\\ -\frac{\nu_{\text{PDMS}}}{E} & \frac{1}{E} & -\frac{\nu_{\text{PDMS}}}{E}\\ -\frac{\nu_{\text{PDMS}}}{E} & -\frac{\nu_{\text{PDMS}}}{E} & \frac{1}{E} \end{array}\right]\left[\begin{array}{c} \sigma_{\text{xx}}\\ 0\\ 0 \end{array}\right], \end{array}$$

and

(4c) $$\begin{array}{l} \varepsilon_{\text{xx},\text{GT}}=\frac{\sigma_{\text{xx}}}{E}. \end{array}$$

Poisson's ratio is defined as the ratio between the axial strain and lateral strain:

(4d) $$\begin{array}{l} \nu_{\text{PDMS}}=-\frac{\varepsilon_{\text{zz},\text{GT}}}{\varepsilon_{\text{xx},\text{GT}}}, \end{array}$$

where ν_*PDMS*_ = 0.5 refers to the Poisson's ratio of PDMS. In combination with a macroscopic estimate of the lateral strain according to:

(4e) $$\begin{array}{l} \varepsilon_{\text{xx},\text{GT}}\approx \frac{\Delta x}{x_{0}}, \end{array}$$

the ground-truth axial strain ε_*zz,GT*_ relates as follows to the lateral displacement *x*:

(4f) $$\begin{array}{l} \varepsilon_{\text{zz},\text{GT}}={-\nu }_{\text{PDMS}}\cdot \frac{\Delta x}{x_{0}}, \end{array}$$

where *x*_0_ refers to the initial lateral length of the sample, which was ~5 mm. The entire sample had dimensions of ~5 × 5 × 5 mm. Lateral displacement was incremented nearly logarithmically from 3.85 to 38.5 μm using a discrete piezoelectric stack actuator (38.5 μm stroke length; Thorlabs Inc., Dachau/Munich, Germany). This displacement range corresponds to an axial strain range of 0.385–3.85‰, which matches well with the observed strain amplitude observed in corneal tissue (0.00–5.11‰). For statistical analysis, six arbitrary locations within the B-scan of a given lateral deformation were selected, which were spaced by more than the accumulated processing windows applied for phase and strain computations. The same relative locations were then tracked across B-scans taken at different lateral deformations. Mean values and standard deviation were calculated to determine strain measurement precision at a single pixel, which in turn was used for comparison with the experimentally determined interspecimen standard deviation in porcine corneas. These parameters were independent of measurement location.

### Estimation of the Stress–Strain Relationship

The stress distribution imposed by the IOP does not have a homogeneous distribution. Assuming that the eye is a thick-walled spherical pressure vessel, the radial σ_r_ and circumferential σ_θ_ = σ_φ_ stress components (Schneider and Kienzler, 2017) expressed in spherical coordinate system (*r*, θ, φ) are given by the Lame's equation:

(5) $$\begin{array}{l} \sigma_{\text{r}}\left(r\right)=\frac{P_{\text{iop}}r_{\text{post}}^{3}-P_{\text{atm}}r_{\text{ant}}^{3}}{\left(r_{\text{ant}}^{3}-r_{\text{post}}^{3}\right)}-\frac{(P_{\text{iop}}-P_{\text{atm}})\cdot r_{\text{post}}^{3}\cdot r_{\text{ant}}^{3}}{(r_{\text{ant}}^{3}-r_{\text{post}}^{3})r^{3}}\text{ and}\\ \sigma_{\text{θ}}(r)=\sigma_{\text{φ}}(r)=\frac{P_{\text{iop}}r_{\text{post}}^{3}-P_{\text{atm}}r_{\text{ant}}^{3}}{\left(r_{\text{ant}}^{3}-r_{\text{post}}^{3}\right)}+\frac{(P_{\text{iop}}-P_{\text{atm}})\cdot r_{\text{post}}^{3}\cdot r_{\text{ant}}^{3}}{2(r_{\text{ant}}^{3}-r_{\text{post}}^{3})r^{3}} \end{array}$$

where *r*_post_ represents the posterior radius of the curvature, *r*_ant_ is the anterior radius of the curvature, *P*_iop_ is the IOP, *P*_atm_ is the atmospheric pressure, and r is the radial distance from the origin. *P*_atm_ was set to zero, as IOP is defined as the pressure difference between IOP and extraocular pressure.

In corneal tissue, ~250 collagen lamellae (Meek, 2009) are stacked one over another, each running from limbus to limbus. Therefore, in approximation, the tissue can be considered being transversely isotropic, with distinct properties in the plane of collagen lamellae compared to their orthogonal direction. Spherical coordinates are used to describe this problem, with the origin located at the center of the eye globe. Furthermore, considering that with IOP changes, only hydrostatic (and no shear) stress is induced and that the eye is approximately spherical, the full three-dimensional (3-D) strain tensor reduces to:

(6a) $$\begin{array}{l} \varepsilon =\left[\begin{array}{ccc} \varepsilon_{\theta \theta } & \varepsilon_{\theta \varphi } & \varepsilon_{\theta \text{r}}\\ \varepsilon_{\varphi \theta } & \varepsilon_{\varphi \varphi } & \varepsilon_{\varphi \text{r}}\\ \varepsilon_{\text{r}\theta } & \varepsilon_{\text{r}\varphi } & \varepsilon_{\text{rr}} \end{array}\right]\approx \left[\begin{array}{ccc} \varepsilon_{\theta } & 0 & 0\\ 0 & \varepsilon_{\theta } & 0\\ 0 & 0 & \varepsilon_{\text{r}} \end{array}\right]. \end{array}$$

Assuming circumferential symmetry ε_θ_ = ε_ϕϕ_ = ε_θθ_ and ε_r_ = ε_rr_, Hook's law for a transversely isotropic material (Lubarda and Chen, 2008) can be written as:

(6b) $$\begin{array}{l} \left[\begin{array}{c} \varepsilon_{\theta }\\ \varepsilon_{\theta }\\ \varepsilon_{\text{r}} \end{array}\right]=\left[\begin{array}{ccc} \frac{1}{E_{\theta }} & -\frac{\nu_{\theta }}{E_{\theta }} & -\frac{\nu_{\theta \text{r}}}{E_{\text{r}}}\\ -\frac{\nu_{\theta }}{E_{\theta }} & \frac{1}{E_{\theta }} & -\frac{\nu_{\theta \text{r}}}{E_{\text{r}}}\\ -\frac{\nu_{\theta \text{r}}}{E_{\theta }} & -\frac{\nu_{\theta \text{r}}}{E_{\theta }} & \frac{1}{E_{\text{r}}} \end{array}\right]\left[\begin{array}{c} \sigma_{\theta }\\ \sigma_{\theta }\\ \sigma_{\text{r}} \end{array}\right], \end{array}$$

where E_θ_ and E_r_ represent the elastic moduli circumferentially along the corneal surface (i.e., the plane of collagen lamellae) and radially (i.e., along corneal thickness, orthogonally to the former), respectively; ν_θ_ and ν_θ*r*_ represent Poisson's ratio, respectively, circumferentially and radially. Applied to the current experimental setting, the following equation is relevant:

(6c) $$\begin{array}{l} \varepsilon_{\text{r}}=\frac{1}{E_{\text{r}}}\sigma_{\text{r}}-\frac{{2\nu }_{\theta \text{r}}}{E_{\theta }}\sigma_{\theta }. \end{array}$$

For typical (Garner et al., 1997) corneal curvature values (*r*_post_≈ 6.4 mm, *r*_ant_≈ 7.2 mm), σ_θ_ dominates over σ_r_. However, the circumferential E-modulus (*E*_θ_) in the direction of the collagen lamellae is considerably higher than the radial E-modulus (*E*_*r*_) along corneal thickness. A recent study (Hatami-Marbini and Etebu, 2013) on porcine eyes reported that the circumferential E-modulus was about three orders of magnitude larger than the radial E-modulus (5.61 kPa vs. 1.33 MPa). Hence, both the terms in Equation 6c are expected to contribute to the experimentally recorded strain maps.

With the current experimental design, bulk motion during an inflation–deflation cycle has only negligible effects on the calculation of stress. Maximal experimentally observed axial bulk displacement was ~25 μm. Compared to typical (Garner et al., 1997) corneal radii of curvature, this amount is <2‰, and thus its effect on stress estimation is very low.

### Statistical Analysis

To evaluate corneal strain distribution as a function of depth, the strain induced by a single step of 1 mmHg IOP increase or decrease, respectively, was averaged within the central region (width ~4 mm) and across five subsequent pressure steps for each individual sample. To assess axial apex displacement, vertical displacements in the central region were averaged across the entire corneal depth for each sample. We also computed the maximal strain amplitude and hysteresis for each sample. A paired two-tailed Student's *t*-test was performed for statistical comparison between 15 and 25 mmHg initial IOP. Similarly, a paired *t*-test was applied to analyze differences in maximal vertical displacement and in the residual vertical displacement, that is, displacement that did not recover after IOP decreased to its initial value. Paired testing is justified since the IOP cycles starting at 15 and 25 mmHg were performed subsequently on the same eye. *P* < 0.05 were considered to indicate significant differences. Pearson correlation was computed to quantify the correlation between axial strain and corneal thickness, as well as postmortem time. Correlation was considered significant with a two-tailed significance of <0.050.

## Results

**Figure 2:**
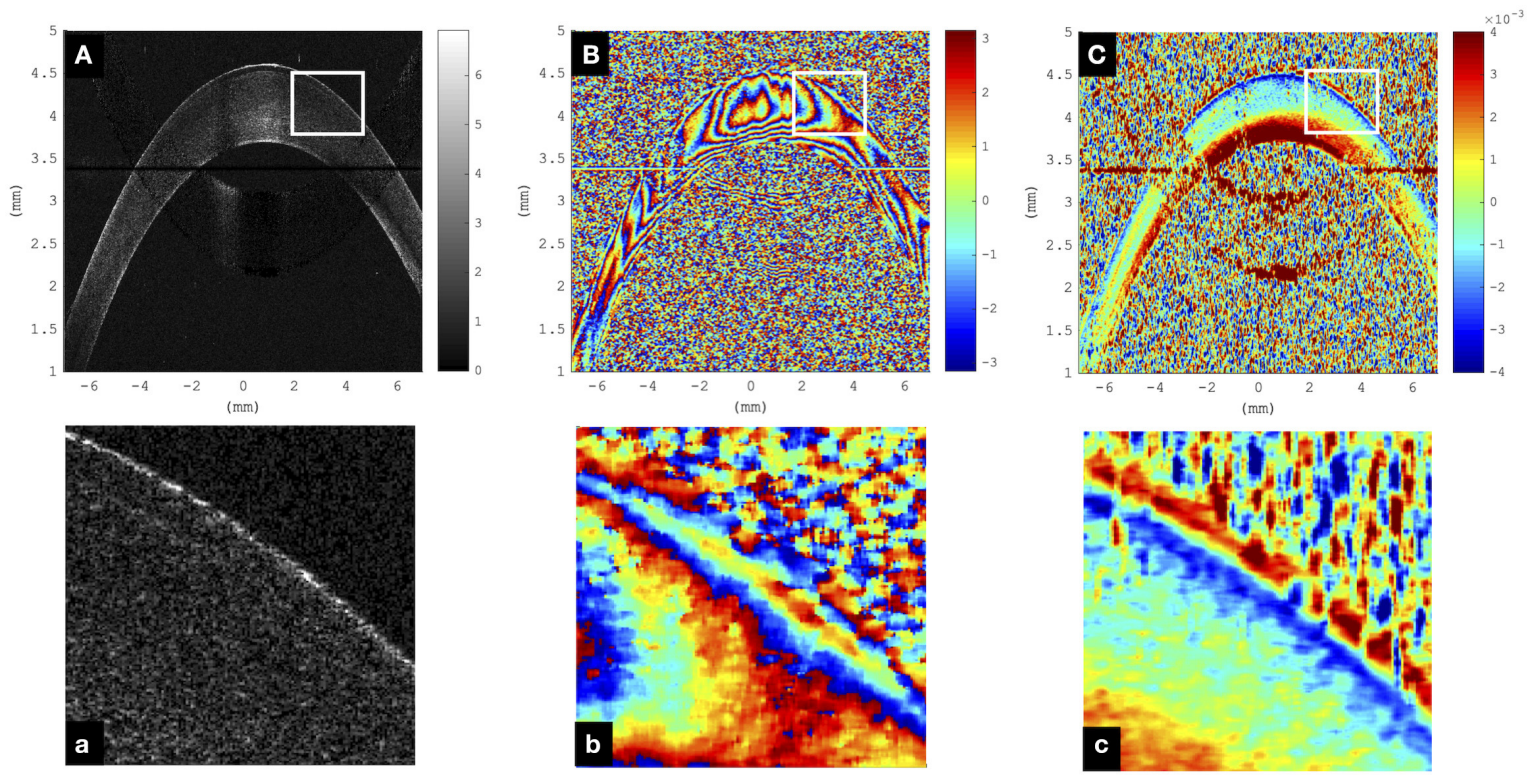
Representative cornea subjected to a single step of 1-mmHg intraocular pressure (IOP) increase. **(A)** Morphological image; the epithelium can only be resolved in the very central region and not in the periphery. Color bar represents intensity in log scale. **(B)** Phase difference map; the epithelium presents as a narrowly spaced phase wrap. Color bar represents phase change in radians. **(C)** Axial strain map; the epithelium can be resolved both in the center and periphery. Substantial signal loss was observed in the region where the cornea overlapped with the frequency-wrapping artifact (mirror image) inherent to Fourier-domain optical coherence tomography (OCT). Color bar represents strain (no unit). **(a–c)** Enlargements of the areas indicated by the white rectangle.

Figure 2 visualizes a comparison of a representative morphological image, corresponding phase difference map, and retrieved axial strain map in porcine eyes. Even though the mirror image could be well-removed in the morphological and phase difference images, artifacts related to the mirror image partially reappeared in the strain image. Interestingly, in the morphological image, the epithelium could only be resolved in the very center, whereas in the strain image, it seemed to be detectable even in the periphery. The epithelial layer accounts for ~10% of corneal thickness (~90–100 μm). Considering the size of the windows applied during phase and strain processing, the detectable thickness is reduced by 6 pixels (~19.6 μm) and 16 pixels (~52.1 μm), respectively. Provided that sufficient backscattering signal is present, hence epithelium is unambiguously identifiable in phase maps but may be masked by artifacts in strain maps. Here, negative strain values indicate compressive strain, and positive values indicate tensile strain. Accordingly, the largest compressive strains were observed in the anterior cornea, whereas the posterior cornea experienced a positive strain.

### Axial Strain Sensitivity

**Figure 3:**
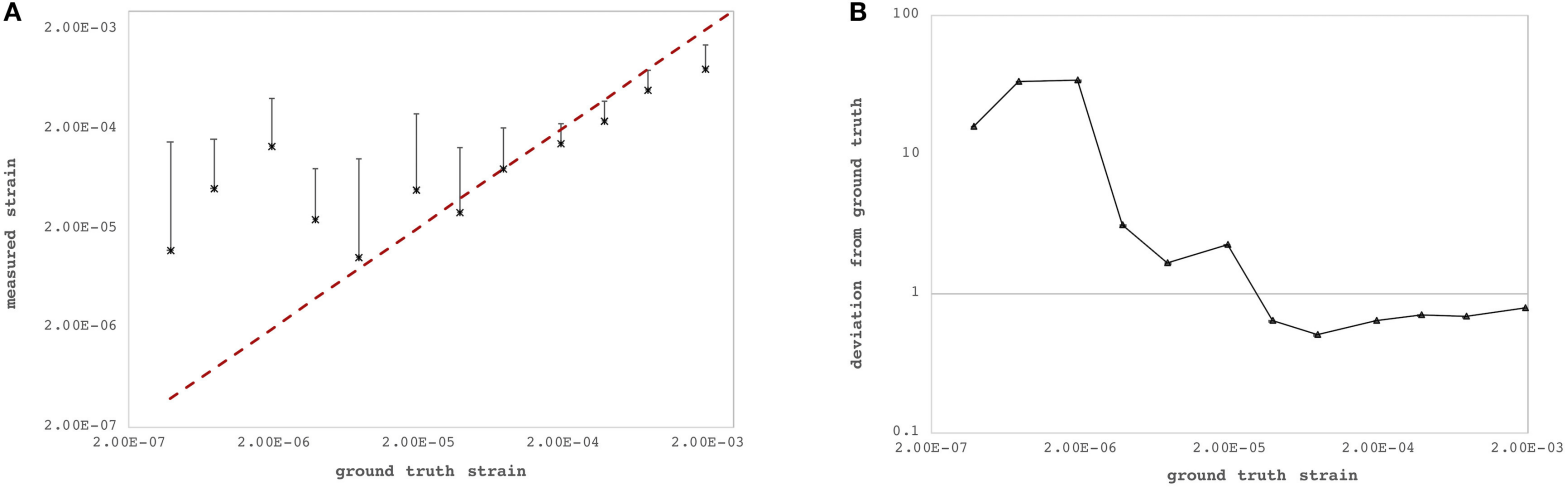
Strain sensitivity across the six individual measurement locations within the polydimethylsiloxan (PDMS) sample. **(A)** Ground-truth strain (red dashed line) vs. measured strain by the proposed optical coherence tomography (OCT)-based method. **(B)** Relative deviation from ground-truth strain. Precision increased with larger strains.

The axial and lateral strain resolutions of OCT measurements depend on the applied processing windows and currently amount to 22 pixels (~71.7 μm) and 7 pixels (~98.0 μm), respectively. Figure 3 summarizes axial strain precision and relative deviation |ε_zz,*GT*_ − ε_zz,exp_|/ε_zz,*GT*_, where ε_zz,exp_ represents the experimentally determined axial strain value for different levels of lateral deformation of the PDMS sample. Relative deviation from ground truth was lower for larger deformations. For strains larger than ~2.44·10^−5^, the OCT-derived strain did match acceptably well with the ground-truth strain (Figure 3B) imposed by the lateral compression and computed by Equation 4f. There was no upper limit of detectable strain within the investigated range of up to 3.85‰ induced axial strain. Because of logarithmic representation, absolute strain values are presented in Figure 3A—but actually, axial strain had a positive sign indicating axial (vertical) expansion during lateral compression, which is typical and expected for isotropic materials such as PDMS. Across the six arbitrary points within the PDMS sample, the standard deviation of strain measurements at a pixel was 0.30‰.

### Corneal Axial Strain

**Figure 4:**
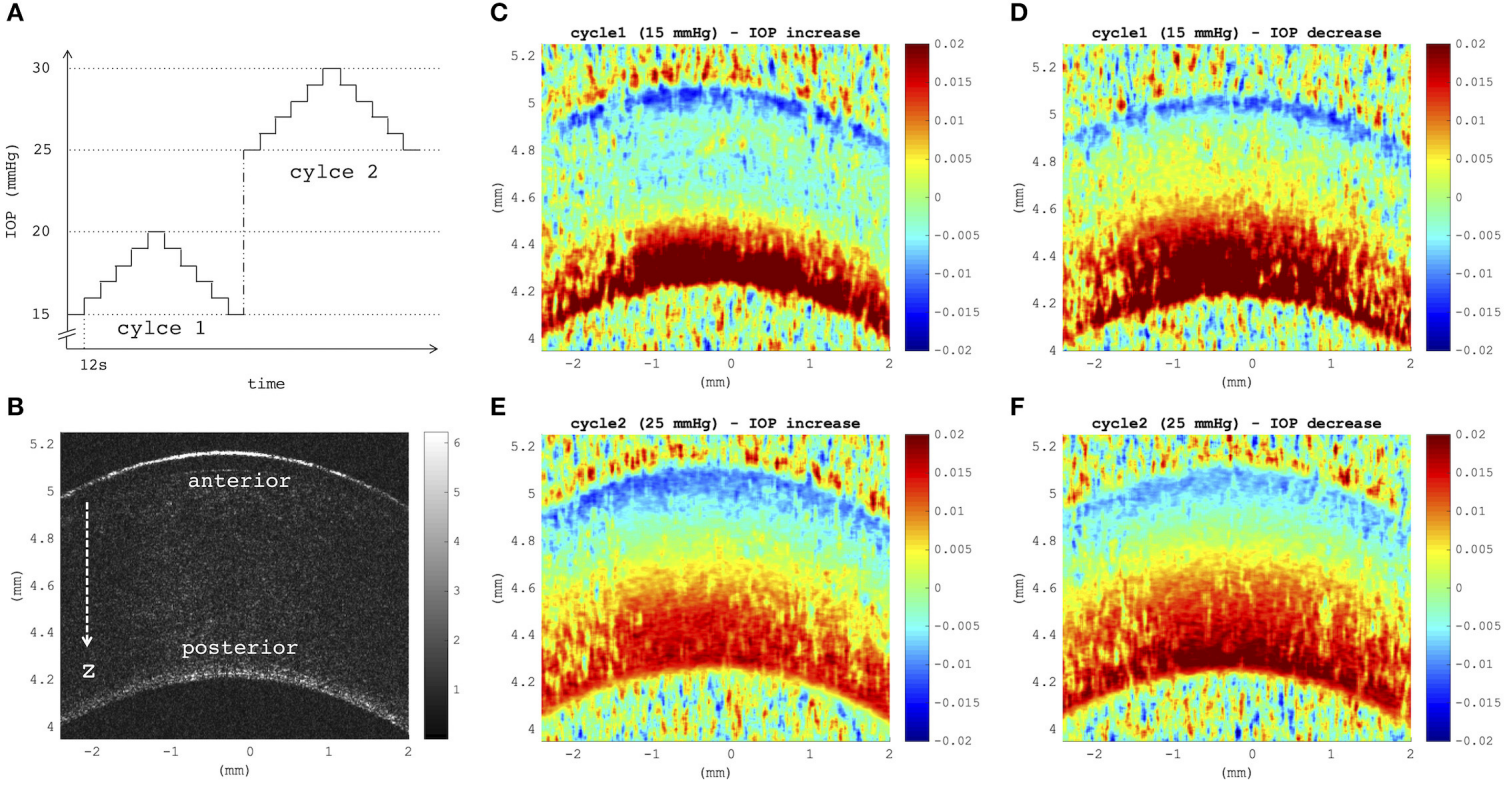
**(A)** Intraocular pressure (IOP) modulation sequence consisting of two subsequent cycles of pressure increase/decrease with reference to initial IOPs of 15 mmHg (cycle 1) and 25 mmHg (cycle 2). **(B)** B-mode morphological image indicating anterior and posterior corneal surfaces, as well as the axial direction *z* of optical coherence tomography (OCT) imaging. **(C–F)** Axial strain induced by a variation of 5 mmHg, obtained by summing over strains retrieved from five subsequent pressure steps of 1 mmHg **(C,E)** during eye inflation and **(D,F)** during deflation. Strain maps at a lower initial IOP showed a larger tension of the posterior stroma than at a higher initial IOP (**D** vs. **F**). Posterior tension was higher during IOP decrease than during increase, independent of the initial IOP. Postmortem time at the time of measurement was ~3 h + 3 h:38 min.

**Figure 5:**
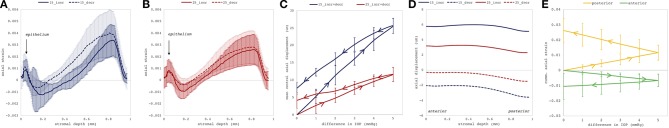
Deformation analyses in the central cornea region. Mean axial strain profiles (*N* = 6 eyes) along corneal thickness in response to a 1-mmHg change in intraocular pressure (IOP), with **(A)** 15 and **(B)** 25 mmHg initial IOP. The largest compressive strains were observed in the posterior stroma. Error bars represent the standard deviation across the six eyes. **(C)** Induced suprapixel bulk displacement of the whole cornea, accumulated for the entire IOP modulation cycle. Error bars represent the standard deviation across the six eyes. **(D)** Combined axial displacement profile consisting of integrated axial gradient and suprapixel displacement for a single 1-mmHg IOP step. **(E)** Cumulative corneal strain in the anterior and posterior corneas as a function of IOP with initial IOP of 15 mmHg. Here, anterior cornea was defined as the region experiencing negative strains and posterior cornea as the region experiencing positive strains. _incr: IOP increase, _decr: IOP decrease, 15: 15 mmHg initial IOP, 25: 25 mmHg initial IOP. Error bars are omitted for clarity.

**Figure 6:**
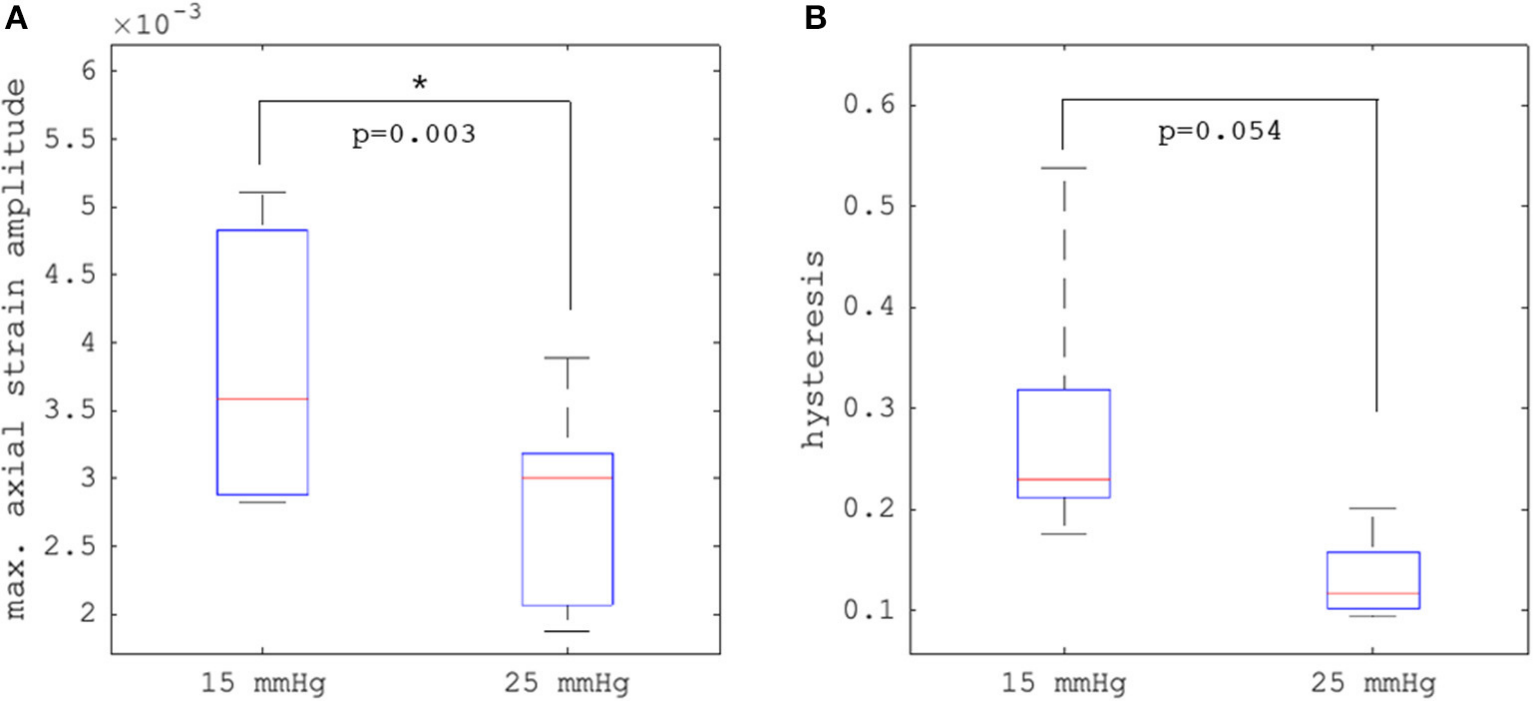
Comparison (*N* = 6 eyes) of **(A)** maximal axial strain amplitude and **(B)** hysteresis between 15 and 25 mmHg initial intraocular pressure (IOP). Error bars represent the standard deviation across the six eyes.

Figures 4C–F depict axial strain maps of a cornea undergoing IOP modulation at different starting IOPs (Figure 4A) in a central region of ~4 mm width (Figure 4B). There was a clear difference in strain distribution between IOP increase and decrease. Most positive strains were induced in the posterior stroma during IOP decrease, particularly with low initial IOP (15 mmHg). Overall, strain amplitudes were lower for an initial loading of 25 mmHg IOP. Figures 5A,B present the axial strain distribution as a function of corneal thickness, averaged across all specimens. The strain distribution of each individual specimen was determined by averaging the corneal strain profiles along the corneal thickness obtained from five subsequent measurement steps (each separated by 1 mmHg IOP change) during the increase and decrease, as well as with 15 and 25 mmHg initial IOP. Error bars represent the standard deviation across the six eyes tested. The mean standard deviation was 0.70‰. The observed strain pattern highlights a strain gradient across corneal thickness. Contrary to what may be expected from Equation 5, positive strain values were observed in the posterior cornea, indicating axial expansion. Figure 5C presents the corresponding bulk suprapixel displacements, which were larger with a lower initial IOP. Figure 5D visualizes the combined axial displacement profile, that is, the integrated strain along corneal thickness combined with the bulk suprapixel displacement of the whole cornea. During the IOP increase, the anterior surface moved more upward than the posterior surface; during the IOP decrease, the posterior surface recovered more than the anterior surface did. Supplementary Figure 1 shows a more detailed analysis of the anterior, central, and vertical posterior corneal displacements. Particularly at 25 mmHg initial IOP, the posterior cornea moved significantly less than the anterior (*p* < 0.001) and central (*p* = 0.016) corneas did in response to IOP modulation and presented a residual displacement of the opposite sign than the anterior (*p* = 0.004) and central (*p* = 0.015) cornea. Figure 5E presents the stress–strain curve in the anterior and posterior corneas. Here, the anterior cornea was defined as the region experiencing negative strains and posterior cornea as the region experiencing positive strains. The accumulated strain was obtained from averaging strains in the anterior or posterior regions, respectively, and summing over subsequent pressure steps. Interestingly, and contrary to vertical apex displacement (in panel C), the corneal strain continued to increase, even when the applied stress (IOP) was reduced. Panel D suggests that this strain behavior results from the anterior cornea being displaced more than the posterior cornea during IOP increase and recovering less during the IOP decrease. Maximal strain amplitudes were observed in the posterior stroma. At a lower initial IOP, maximal strain amplitude was significantly (*p* = 0.003) larger (3.80 ± 0.98‰) compared to a higher initial IOP (2.48 ± 0.75‰) (Figure 6A).

### Hysteresis

Hysteresis was defined as the area enclosed in the axial strain profile between the IOP increase and decrease curves. Corneal hysteresis showed a trend toward larger values at 15 mmHg compared to 25 mmHg (0.283 ± 0.133 vs. 0.131 ± 0.041, *p* = 0.054) (Figure 6B). Table 1 provides further details on the statistical comparisons performed.

**Table 1:** Individual deformation values of each specimen along with *p*-values obtained from the two-tailed paired Student's *t*-test.

**Eye n°**	**Time**	**Max. strain amplitude (‰)**		**Hysteresis**		**Anterior IOP increase (‰)**		**Posterior IOP increase (‰)**		**Anterior IOP decrease (‰)**		**Posterior IOP decrease (‰)**	
	**hh:mm**	**15 mmHg**	**25 mmHg**	**15 mmHg**	**25 mmHg**	**15 mmHg**	**25 mmHg**	**15 mmHg**	**25 mmHg**	**15 mmHg**	**25 mmHg**	**15 mmHg**	**25 mmHg**
1	00.00	2.83	2.07	0.17	0.16	−0.02	−0.11	2.56	2.06	0.91	0.84	3.31	2.25
2	00:31	2.88	1.87	0.54	0.12	−1.59	−0.37	1.93	1.58	−0.52	−0.23	3.97	2.28
3	01:21	3.31	2.95	0.24	0.11	−2.49	−0.97	2.72	2.83	−1.67	−0.42	4.04	3.20
4	03:38	4.83	3.18	0.21	0.09	−1.71	−1.69	5.27	2.89	−1.47	−1.38	4.55	3.49
5	04:07	5.11	3.89	0.32	0.10	−2.31	−2.26	4.97	3.78	−1.76	−1.75	5.37	4.03
6	06:02	3.86	3.05	0.22	0.20	−3.01	2.20	4.22	3.48	−1.85	−1.19	3.62	2.66
*p*	–	0.003		0.054		0.091		0.063		0.129		<0.001	

*IOP, intraocular pressure*.

### Postmortem Time

**Figure 7:**
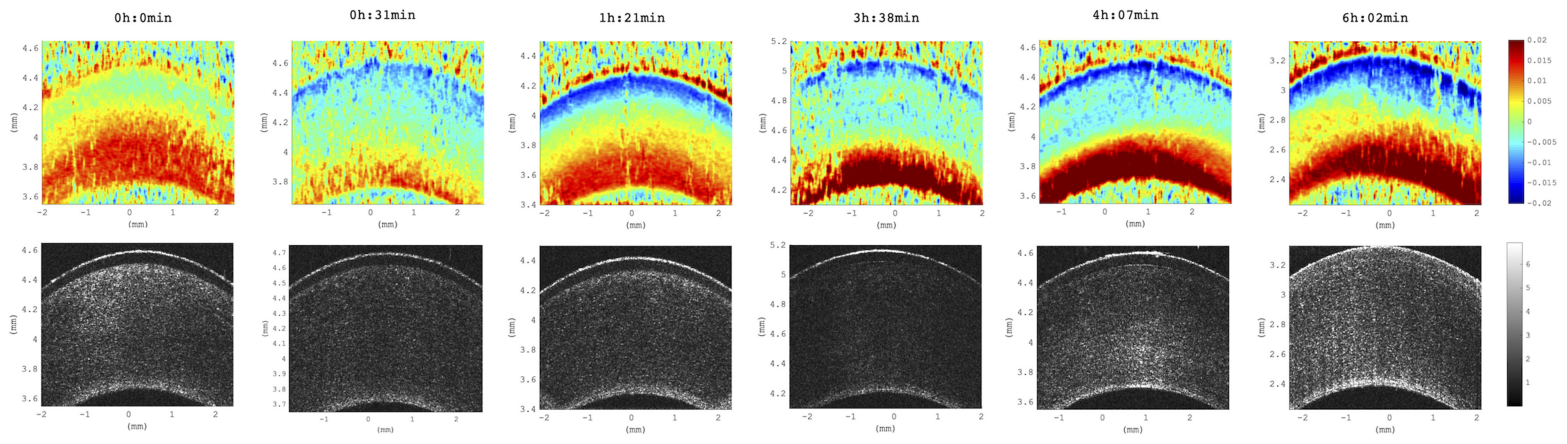
Axial strain maps as a function of increasing postmortem time. Distribution across all six eyes studied. Time references to the beginning of the experiment, at which porcine eyes were ~3 h postmortem. Strain amplitude and signal strength increased with postmortem time across all samples included in this study (*N* = 6 eyes).

**Figure 8:**
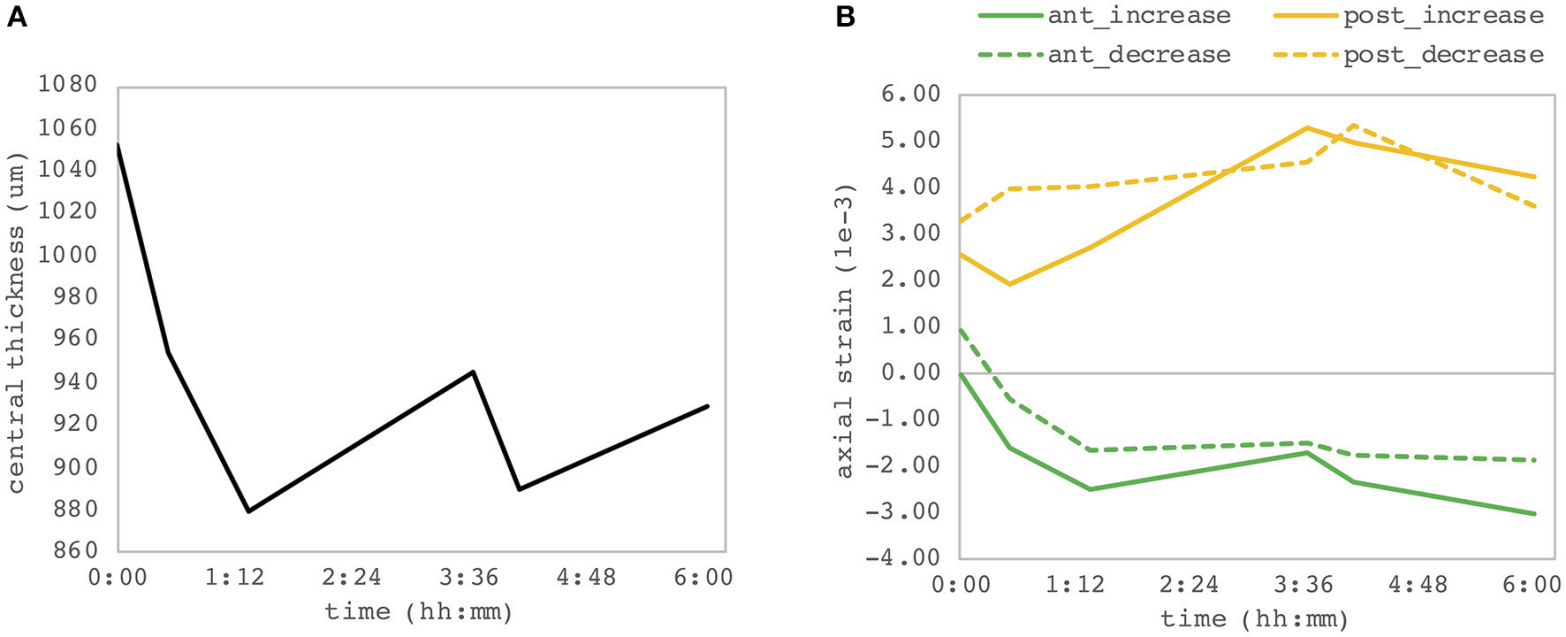
Dependency on postmortem time. **(A)** Central corneal thickness and **(B)** maximal corneal strain in the anterior and posterior corneas. Each of the six eye samples contributes as one point of the individual curves. A significant correlation between the anterior corneal strain and both postmortem time and corneal thickness was observed.

We observed a substantial change of axial strain amplitude and distribution with increasing experimental time and hence with longer postmortem time. Figure 7 shows a time series of axial strain maps retrieved from subsequently measured eyes with different postmortem times. A clear trend of increased posterior tensile strain and anterior compressive strain can be observed with increasing postmortem time. Interestingly, also signal strength seemed to improve with time, whereas epithelial visibility in the morphological images degraded with time. Along with epithelial vanishing in the morphological images, it became more apparent in the strain maps likely as a result of increased optical scattering. Figure 8 presents the evolution of corneal thickness and maximal strain values in the anterior and posterior corneas. The thinner the corneal tissue, a trend toward larger strain amplitudes was observed. In particular, anterior strain was significantly correlated with corneal thickness at 15 mmHg (c_pearson_ = 0.917, *p* = 0.010) but not at 25 mmHg (c_pearson_ = 0. 706, *p* = 0.117). Anterior strain also correlated significantly with postmortem time at 25 mmHg (c_pearson_ = 0.910, *p* = 0.012) but not at 15 mmHg (c_pearson_ = 0. 759, *p* = 0.080). A significant correlation of corneal strain between 15 and 25 mmHg was observed, both in the anterior (c_pearson_ = −0.837, *p* = 0.038) and posterior (c_pearson_ = −0.837, *p* = 0.038) tissues. In contrast, posterior strain was not significantly affected by postmortem time or corneal thickness.

## Discussion

In this study, the mechanical response of corneal tissue was investigated in response to low-amplitude IOP changes that are comparable to physiologic diurnal IOP fluctuations (David et al., 1992). It was demonstrated that phase-sensitive OCT has sufficient sensitivity to resolve axial strain distribution in the corneal tissue resulting from IOP changes of as little as 1 mmHg. This implies that corneal biomechanical properties can be investigated by inducing 10 to 10^4^ times lower stresses than those described before in extensometry (Zeng et al., 2001) (~3 MPa ≈ 22,500 mmHg), air-puff tonometry (Kling et al., 2014) (~110 mmHg), or quasi-static compression-based OCE approaches (De Stefano et al., 2018) (~15–20 mmHg). Lower test loads permit material characterization within physiological conditions. Hence, the retrieved mechanical properties are likely more relevant for clinical prognosis and diagnosis and more valuable as input for numerical simulations (Alastrué et al., 2006; Sinha Roy and Dupps, 2009; Whitford et al., 2018). In addition to a more natural stress amplitude, the applied stress distribution did resemble physiological conditions in the current setup, which is in contrast to most previous studies investigating corneal biomechanics. Recently, digital volume correlation on 3-D OCT scans (Fu et al., 2016) was applied to track corneal deformations induced by a similar range of IOP modulation (15–18.85 mmHg). This approach however is limited in spatial resolution and less sensitive to subpixel deformation. Compared to non-linear optical imaging techniques, such as Brillouin microscopy (Scarcelli et al., 2014, 2015), which does not require any kind of mechanical deformation to assess elastic material properties, the presented approach can be interpreted as a more direct measure. OCE is able to straightforwardly extract mechanical strain values. In contrast, Brillouin microscopy relies on a frequency shift in backscattered light, which only correlates with mechanical (and other) parameters. Axial strain maps quantify actual sample deformation and therefore are directly linked to corneal geometry and thus refractive properties. Therefore, strain maps could arguably be even more predictive in terms of clinical refractive stability than quantitative metrics, such as e-moduli, since strain would be sensitive to the interaction between relative (i.e., local) material differences. Interestingly, bulk motion did not prevent strain and hence phase difference analysis, as long as the displacement was macroscopically corrected by shifting the reference image by the corresponding amount of pixels.

Strain deviation from nominal values was lower than 100% for strains larger than 2.44 · 10^−5^, which we consider acceptable for the current application. A deviation of <100% implies that changes in the sign of strain can be correctly identified. Better agreement with ground-truth values for higher strains (Figure 2) was expected due to the specific design of the experiment. Ground-truth strains were chosen to include strain two orders of magnitude lower than the theoretically expected threshold for minimally detectable strain: Precision of strain measurements depends largely on the phase stability of the light source. Typical values for superluminescent diodes are 0.48 mrad corresponding to a displacement sensitivity of 34 pm (Zhang et al., 2009). Strain is computed by taking the axial resolution into account. Hence, for an axial resolution of 3.26 μm in tissue, the corresponding strain sensitivity amounts to 1.53 · 10^−5^, which is very close to the actual determined minimally detectable strain of the current study.

The strength of the OCT-based strain estimation method lies in the spatial resolution of the retrieved strain maps:the standard deviation of strain measurement at a single pixel was 0.30‰, which is by factor 10 less than the maximal strain amplitudes (3.96‰) observed in corneal tissue and by factor 2 less than the interspecimen standard deviation (0.70‰) encountered. Spatial resolution of strain maps is currently limited to 71.7 μm axially and 98.0 μm laterally. Further refinement of processing windows, for example, by using an adaptive windowing approach, could locally increase strain resolution and improve epithelial strain retrieval. Without this tool, interpretation of structures of dimensions exceeding 20 μm axially and laterally can be performed on the phase map, which however is less intuitive. Nonetheless, a distinct sign of strain of epithelium and anterior stroma can be recognized by visual inspection of the phase map, that is, by taking the vertical gradient, in Figure 2B,b. In this regard, the proposed corneal elastography approach may also be useful for segmentation in future applications. Some degree of artifact strain was observed in the strain images (Figures 1C, 2C–F) that do not have a morphological or functional meaning. These localized strain fluctuations arise from noise in acquired OCT scans. Applying larger processing windows could reduce such artifacts, however, at the same time would reduce the image resolution. Future studies in diseased corneas shall shed further light on the required strain resolution for diagnostic purposes. For the interpretation of results in the current study, artifact strains play a negligible role for corneal strain profile and hysteresis given that these analyses were performed on mean strain values across the central cornea, in which artifact strains are averaged out.

A significantly larger strain amplitude and larger hysteresis were observed at the lower compared to the higher initial IOP. The stress–strain curve (Figure 5E) did show a linear relation, with the slope indicating material stiffness (elastic modulus). Previously, corneal tissue has been described as a non-linear material (Zeng et al., 2001), with increasing stiffness at larger strains. Yet, these measurements were performed at much larger strains, and the non-linear behavior is likely less prominent within physiological strain levels. The extent of material non-linearity and the suitability of using such material models in a small strain regime for corneal tissue in numerical simulations (Alastrué et al., 2006; Sinha Roy and Dupps, 2009; Whitford et al., 2018) should be further evaluated in future studies. In terms of clinical relevance, particularly with respect to the diagnosis of glaucoma, the sensitivity of strain amplitude and hysteresis toward IOP is even more pertinent than actual corneal stiffness, as for the latter, the stress distribution within the tissue needs to be known, which may vary between individuals. Indentation-based techniques (air-puff, anterior compression) assess the absolute tissue displacement as a function of applied load. However, it is difficult to distinguish between increased IOP and stiff corneal tissue. Moreover, within physiological ranges, IOP has a larger effect than mechanical properties (Kling and Marcos, 2013), which makes it particularly difficult to analyze the latter with air-puff deformation. So far, clinical studies did not find a significant difference after corneal cross-linking with air-puff measurements (Goldich et al., 2009). In the current study, it was shown that the mean displacement, that is, bulk motion, for a loading step (Figure 5C) was 2.2 times larger at 15 mmHg compared to 25 mmHg. Bulk motion in the current setup refers to the axial motion of corneal tissue within the imaging borders. However, given that the eye was fixed in a customized hemispherical silicon mold, any bulk motion of the entire eye globe can be excluded. Therefore, vertical corneal bulk motion represents the increase in ocular diameter and as thus allows for assessing the amount of eye globe expansion. As expected, the observed vertical bulk motion suggested only a minor change in ocular diameter (<2‰) such that corneal stress distribution is only negligibly changed during the entire IOP cycle.

A strain gradient across corneal thickness was found, with largest strain amplitudes (i.e., absolute strain values) being observed in the posterior stroma and lower strains toward the anterior stroma (Figures 5A,B). Such a strain gradient meets our expectations based on the stress gradient in a thick-walled pressurized spherical membrane (Equation 5). Surprisingly, positive strains were observed in the posterior stroma, indicating axial tension. This unexpected observation, which is somewhat counterintuitive based on the expectations of a simple mechanical behavior, might be a result of the particular strain distribution in the cornea and could indicate unique material properties, for example, auxetic material behavior, that is, negative Poisson's ratio, which leads to expansion in the orthogonal direction when being stretched. Auxetic behavior on collagen fibril level has previously been observed in x-ray crystallography when corneal tissue was subjected to tension (Patten and Wess, 2013), even though macroscopically, the tissue showed positive Poisson's ratio (Bell et al., 2018). Macroscopic analysis might have masked localized strain oppositions, which might be accessible with the resolution of the OCT setup herein. Auxetic behavior of corneal tissue however cannot be confirmed definitely with the results presented herein. To accurately interpret the role of depth-resolved Poisson's ratio in corneal tissue, uniaxial corneal characterization is required, which is beyond the scope of this study. An alternative explanation to this unexpected biphasic strain distribution is osmotic pressure changes and related diffusion gradients in and around the cornea. Cornea *in situ* is a strongly hydrated tissue and has a swelling pressure (Dohlman et al., 1962) of ~55–60 mmHg. It is reasonable to assume that the swelling capacity of corneal tissue increases with postmortem time because of endothelial cell death. Given that for the purpose of this study only small IOP loads (maximum 30 mmHg) were applied, corneal swelling could have dominated in the posterior section, where it was directly in contact with the aqueous humor. In contrast, the anterior part had less capacity to swell and thus the compressive force imposed by the IOP modulation would dominate. Hence, this may indicate that biphasic corneal strain distribution is a peculiarity of *ex vivo* tissue.

The observation that corneal strain, both in the anterior and posterior corneas, continued to increase even when the applied stress (IOP) was released corroborates the above hypothesis. Although the vertical apex displacement recovered after reducing the IOP, the osmotic swelling could have further progressed and potentially caused the anterior compression. To estimate the extent of osmotic swelling during the measurement, consider the following: When storing enucleated porcine eyes for 24 h (without IOP control), they typically swell by ~300 μm, corresponding to a rate of 3.5 nm/s, assuming swelling at a constant speed. A similar speed (3.6 nm/s) is obtained when computing the slope of corneal thickness across time in the current experiment. With a temporal separation between subsequent measurement steps of 14 s, an axial strain contribution of 49 nm/800 μm = 0.06‰ can be expected. This strain originating from osmotic swelling is substantially lower than posterior strain values (~1‰). An alternative interpretation to explain that strain continued to increase during pressure decrease considers two additional factors: (1) corneal tissue has viscoelastic properties and (2) enucleated eyes lose IOP with time. Potentially, adjusting the initial IOP to 15 or 25 mmHg could have induced a creep that masked the subsequent pressure modulation. A limitation of the current study arising from these hypotheses is that neither osmotic swelling nor creep could be separated from IOP-induced deformations.

Evidence is provided that OCT strain maps are sensitive to postmortem time (Figure 7), which probably also goes along with an increased swelling pressure in the posterior stroma due to loss of endothelial pump function. In this context, increased OCT signal strength likely arises because of transparency loss going along with increased hydration levels, which in turn lead to a larger proportion of light being backscattered at this wavelength. While this is a limitation of the current *ex vivo* study, at the same time, the strong dependency, particularly of the sign of the induced axial strain, on hydration could be an interesting feature for the diagnosis of Fuchs dystrophy, in which endothelial pump function is impaired (Elhalis et al., 2010).

Note that axial strain maps require careful interpretation: At first sight, the posterior stroma appears weaker because of the largest strain amplitudes. However, this profile is imposed by the applied stress gradient. More sophisticated methods will be required to extract actual stiffness maps from these strain maps. An interesting preliminary observation is that the epithelial layer did experience a distinct deformation than the directly underlying anterior stroma (Figures 2, 5A,B). On the one hand, this confirms that the endothelium may not contribute in counteracting the stress imposed by the IOP (Elsheikh et al., 2008). On the other hand, the opposite deformation behavior could relate to regulation mechanisms of the epithelial cells directed to maintain their shape, which would occur within the “blind time” (12 s) between two subsequent pressure steps.

Axial strain maps as obtained in the current study are a valuable input to finite element simulations of corneal surgeries. Even though these 2-D strain maps cannot provide directional information, they will allow initializing the simulation with a more realistic pre-strain distribution. Currently, pre-stress (instead of pre-strain) conditions resulting from the IOP are considered in finite element simulations (Elsheikh et al., 2013). Nevertheless, the corneal strain distribution resulting from posterior pressure application is compressive in nature, highest at the posterior surface and decreases toward the anterior surface. This study, however, indicates that corneal stroma has regions of positive and negative strains under physiologic loading conditions, which may have important consequences for biomechanical behavior of corneal tissue in simulations. In this line, future studies are envisioned to investigate strain differences in localized pathologies, such as keratoconus, and the effect of corneal stiffening, such as after UV cross-linking treatment (Wollensak et al., 2003).

In the current study, small strains were induced by means of minimal IOP changes. However, this does not restrict OCT-based small strain imaging to be transferred to other areas. In the domain of mechanotransduction, potentially compatible mechano-coupling techniques for subjecting cells, or tissue samples, to microscale mechanical strains have already been developed (Quinn et al., 2002). OCT is a non-contact imaging technique and hence could also be used under sterile conditions to image cellular deformation resulting (Ramage et al., 2009) from an osmotic gradient or in response to adding a biochemical stimulus.

## Data Availability Statement

The raw/processed data required to reproduce these findings are available from the corresponding author on request.

## Author Contributions

SK perceived and designed the study, performed the experiments, processed, analyzed and interpreted the data, drafted the manuscript, and obtained funding. HK adapted the experimental setup, performed the experiments, and revised the manuscript. OG contributed to image processing algorithms, biomechanical interpretation, and revised the manuscript.

### Conflict of Interest

The authors declare that the research was conducted in the absence of any commercial or financial relationships that could be construed as a potential conflict of interest.

## References

[B1] Alastrué V. Calvo B. Peña E. Doblaré M. (2006). Biomechanical modeling of refractive corneal surgery. J. Biomech. Eng. 128, 150–160. 10.1115/1.2132368 16532629

[B2] Andreassen T. T. Simonsen A. H. Oxlund H. (1980). Biomechanical properties of keratoconus and normal corneas. Exp. Eye Res. 31, 435–441. 10.1016/s0014-4835(80)80027-3 7449878

[B3] Ariza-Gracia M. Á. Zurita J. F. Piñero D. P. Rodriguez-Matas J. F. Calvo B. (2015). Coupled biomechanical response of the cornea assessed by non-contact tonometry. A simulation study. PLoS ONE 10:e0121486. 10.1371/journal.pone.0121486 25780915 PMC4364121

[B4] Bayoumi S. (2018). Engineering Solid Mechanics: Fundamentals and Applications. Boca Raton, FL; London; New York, NY; Washington, DC: CRC Press.

[B5] Bell J. S. Hayes S. Whitford C. Sanchez-Weatherby J. Shebanova O. Vergari C.. (2018). The hierarchical response of human corneal collagen to load. Acta Biomater. 65, 216–225. 10.1016/j.actbio.2017.11.015 29128531 PMC5729024

[B6] David R. Zangwill L. Briscoe D. Dagan M. Yagev R. Yassur Y. (1992). Diurnal intraocular pressure variations: an analysis of 690 diurnal curves. Br. J. Ophthalmol. 76, 280–283. 10.1136/bjo.76.5.280 1356429 PMC504256

[B7] De Stefano V. S. Ford M. R. Seven I. Dupps W. J. (2018). Live human assessment of depth-dependent corneal displacements with swept-source optical coherence elastography. PLoS ONE 13:e0209480. 10.1371/journal.pone.0209480 30592752 PMC6310362

[B8] Dohlman C. H. Hedbys B. O. Mishima S. (1962). The swelling pressure of the corneal stroma. Invest. Ophthalmol. Visual Sci. 1, 158–162. 13886960

[B9] Drexler W. Fujimoto J. G. (2008). Optical Coherence Tomography: Technology and Applications. Berlin; Heidelberg: Springer Science and Business Media.

[B10] Elhalis H. Azizi B. Jurkunas U. V. (2010). Fuchs endothelial corneal dystrophy. Ocul. Surf. 8, 173–184. 10.1016/S1542-0124(12)70232-X 20964980 PMC3061348

[B11] Elsheikh A. Alhasso D. Rama P. (2008). Assessment of the epithelium's contribution to corneal biomechanics. Exp. Eye Res. 86, 445–451. 10.1016/j.exer.2007.12.002 18221938

[B12] Elsheikh A. Anderson K. (2005). Comparative study of corneal strip extensometry and inflation tests. J. R. Soc. Interf. 2, 177–185. 10.1098/rsif.2005.0034 16849178 PMC1629069

[B13] Elsheikh A. Whitford C. Hamarashid R. Kassem W. Joda A. Büchler P. (2013). Stress free configuration of the human eye. Med. Eng. Phys. 35, 211–216. 10.1016/j.medengphy.2012.09.006 23041490

[B14] Fu J. Haghighi-Abayneh M. Pierron F. Ruiz P. D. (2016). Depth-resolved full-field measurement of corneal deformation by optical coherence tomography and digital volume correlation. Exp. Mech. 56, 1203–1217. 10.1007/s11340-016-0165-y

[B15] Garner L. F. Owens H. Yap M. K. Frith M. J. Kinnear R. F. (1997). Radius of curvature of the posterior surface of the cornea. Optometry Vision Sci. 74, 496–498. 10.1097/00006324-199707000-00016 9293516

[B16] Goldich Y. Barkana Y. Morad Y. Hartstein M. Avni I. Zadok D. (2009). Can we measure corneal biomechanical changes after collagen cross-linking in eyes with keratoconus?-a pilot study. Cornea 28, 498–502. 10.1097/ICO.0b013e318190734d 19421050

[B17] Hatami-Marbini H. Etebu E. (2013). An experimental and theoretical analysis of unconfined compression of corneal stroma. J. Biomech. 46, 1752–1758. 10.1016/j.jbiomech.2013.03.013 23664313

[B18] Kling S. Bekesi N. Dorronsoro C. Pascual D. Marcos S. (2014). Corneal viscoelastic properties from finite-element analysis of in vivo air-puff deformation. PLoS ONE 9:e104904. 10.1371/journal.pone.0104904 25121496 PMC4133267

[B19] Kling S. Marcos S. (2013). Contributing factors to corneal deformation in air puff measurements. Invest. Ophthalmol. Visual Sci. 54, 5078–5085. 10.1167/iovs.13-12509 23821200

[B20] Kling S. Remon L. Pérez-Escudero A. Merayo-Lloves J. Marcos S. (2010). Corneal biomechanical changes after collagen cross-linking from porcine eye inflation experiments. Invest. Ophthalmol. Visual Sci. 51, 3961–3968. 10.1167/iovs.09-4536 20335615

[B21] Lubarda V. Chen M. (2008). On the elastic moduli and compliances of transversely isotropic and orthotropic materials. J. Mech. Mater. Struct. 3, 153–171. 10.2140/jomms.2008.3.153

[B22] Meek K. M. (2009). Corneal collagen—its role in maintaining corneal shape and transparency. Biophys. Rev. 1, 83–93. 10.1007/s12551-009-0011-x 28509987 PMC5425665

[B23] Patten K. Wess T. (2013). Suprafibrillar structures of collagen, evidence for local organization and auxetic behaviour in architectures. J. Biophys. Chem. 4, 103–109. 10.4236/jbpc.2013.43014

[B24] Quinn T. P. Schlueter M. Soifer S. J. Gutierrez J. A. (2002). Cyclic mechanical stretch induces VEGF and FGF-2 expression in pulmonary vascular smooth muscle cells. Am. J. Physiol. Lung Cell. Mol. Physiol. 282, L897–L903. 10.1152/ajplung.00044.2001 11943652

[B25] Ramage L. Nuki G. Salter D. M. (2009). Signalling cascades in mechanotransduction: cell–matrix interactions and mechanical loading. Scand. J. Med. Sci. Sports 19, 457–469. 10.1111/j.1600-0838.2009.00912.x 19538538

[B26] Sanchez I. Martin R. Ussa F. Fernandez-Bueno I. (2011). The parameters of the porcine eyeball. Graefe's Archive Clin. Exp. Ophthalmol. 249, 475–482. 10.1007/s00417-011-1617-9 21287191

[B27] Scarcelli G. Besner S. Pineda R. Kalout P. Yun S. H. (2015). In vivo biomechanical mapping of normal and keratoconus corneas. JAMA Ophthalmol. 133, 480–482. 10.1001/jamaophthalmol.2014.5641 25611213 PMC4698984

[B28] Scarcelli G. Besner S. Pineda R. Yun S. H. (2014). Biomechanical characterization of keratoconus corneas *ex vivo* with Brillouin microscopy. Invest. Ophthalmol. Visual Sci. 55, 4490–4495. 10.1167/iovs.14-14450 24938517 PMC4109405

[B29] Schneider P. Kienzler R. (2017). Dimensioning of thick-walled spherical and cylindrical pressure vessels. Math. Mech. Solids 2017:1081286517713243 10.1177/1081286517713243

[B30] Shah S. Laiquzzaman M. Bhojwani R. Mantry S. Cunliffe I. (2007). Assessment of the biomechanical properties of the cornea with the ocular response analyzer in normal and keratoconic eyes. Invest. Ophthalmol. Visual Sci. 48, 3026–3031. 10.1167/iovs.04-0694 17591868

[B31] Sinha Roy A. Dupps W. J. (2009). Effects of altered corneal stiffness on native and postoperative LASIK corneal biomechanical behavior: a whole-eye finite element analysis. J. Refractive Surgery 25, 875–887. 10.3928/1081597X-20090917-09 19835328

[B32] Vinciguerra R. Ambrósio R. Elsheikh A. Roberts C. J. Lopes B. Morenghi E.. (2016). Detection of keratoconus with a new biomechanical index. J. Refractive Surg. 32, 803–810. 10.3928/1081597X-20160629-01 27930790

[B33] Wang S. Larin K. V. (2014). Noncontact depth-resolved micro-scale optical coherence elastography of the cornea. Biomed. Opt. Exp. 5, 3807–3821. 10.1364/BOE.5.003807 25426312 PMC4242019

[B34] Whitford C. Movchan N. V. Studer H. Elsheikh A. (2018). A viscoelastic anisotropic hyperelastic constitutive model of the human cornea. Biomech. Model. Mechanobiol. 17, 19–29. 10.1007/s10237-017-0942-2 28780705 PMC5807485

[B35] Wilensky J. T. Gieser D. K. Dietsche M. L. Mori M. T. Zeimer R. (1993). Individual variability in the diurnal intraocular pressure curve. Ophthalmology 100, 940–944. 10.1016/s0161-6420(93)31551-4 8510909

[B36] Wollensak G. Spoerl E. Seiler T. (2003). Riboflavin/ultraviolet-A–induced collagen crosslinking for the treatment of keratoconus. Am. J. Ophthalmol. 135, 620–627. 10.1016/s0002-9394(02)02220-1 12719068

[B37] Zaitsev V. Y. Matveyev A. L. Matveev L. A. Gelikonov G. V. Sovetsky A. A. Vitkin A.. (2016). Optimized phase gradient measurements and phase-amplitude interplay in optical coherence elastography. J. Biomed. Opt. 21:116005. 10.1117/1.JBO.21.11.116005 27824215

[B38] Zeng Y. Yang J. Huang K. Lee Z. Lee X. (2001). A comparison of biomechanical properties between human and porcine cornea. J. Biomech. 34, 533–537. 10.1016/s0021-9290(00)00219-0 11266678

[B39] Zhang J. Rao B. Yu L. Chen Z. (2009). High-dynamic-range quantitative phase imaging with spectral domain phase microscopy. Opt. Lett. 34, 3442–3444. 10.1364/OL.34.003442 19881621 PMC3337213

[B40] Zotter S. Pircher M. Götzinger E. Torzicky T. Bonesi M. Hitzenberger C. K. (2010). Sample motion-insensitive, full-range, complex, spectral-domain optical-coherence tomography. Opt. Lett. 35, 3913–3915. 10.1364/OL.35.003913 21124563 PMC3045030

