# Supplementary material for: Optical Coherence Elastography-Based Corneal Strain Imaging During Low-Amplitude Intraocular Pressure Modulation
Source: Front Bioeng Biotechnol. 2020 Jan 31;7:453. doi: 10.3389/fbioe.2019.00453 (PMC7004960; doi:10.3389/fbioe.2019.00453)
Supplement: Supplementary file 1 [file Image_1.pdf]

Supplementary Material

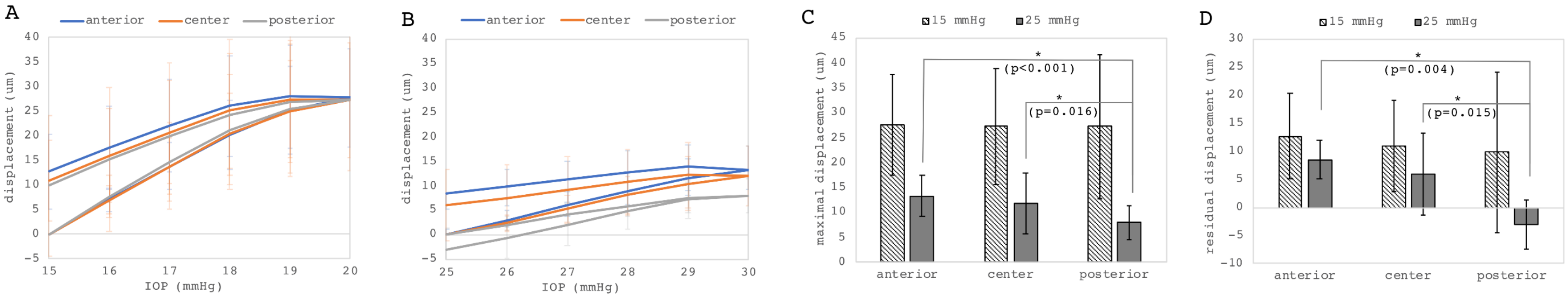

Supplementary Figure 1. Detailed vertical corneal displacement. With (**A**) 15 mmHg, and (**B**) 25 mmHg initial IOP. Comparison of the (**C**) axial displacement at maximal IOP increase, and (**D**) residual vertical displacement, i.e. displacement remaining after IOP decrease to its initial value. Particularly at 25 mmHg, posterior cornea was significantly less displaced than the anterior or central cornea, and presented residual strain of opposite sign. This confirms the observed strain maps indicating that posterior cornea expands and anterior cornea gets compressed.
